# Comparative and phylogenetic analyses of nine complete chloroplast genomes of Orchidaceae

**DOI:** 10.1038/s41598-023-48043-2

**Published:** 2023-12-04

**Authors:** Likuan Liu, Jingxuan Du, Zhihua Liu, Wenming Zuo, Zhenglei Wang, Jinping Li, Yang Zeng

**Affiliations:** 1 College of Life Sciences, Qinghai Normal University, Xining, China; 2 Academy of Plateau Science Sustainability, Xining, China; 3 School of Statistics and Mathematics, Zhongnan University of Economics and Law, Wuhan, China; 4 College of Geosciences, Qinghai Normal University, Xining, China

**Keywords:** Ecology, Plant sciences

## Abstract

The orchid family has 200,000 species and 700 genera, and it is found worldwide in the tropics and subtropics. In China, there are 1247 species and subspecies of orchids belonging to the Orchidaceae family. Orchidaceae is one of the most diverse plant families in the world, known for their lush look, remarkable ecological tolerance, and capability for reproduction. It has significant decorative and therapeutic value. In terms of evolution, the orchid family is one of the more complicated groups, but up until now, little has been known about its affinities. This study examined the properties of 19 chloroplast (cp) genomes, of which 11 had previously been published and nine had only recently been revealed. Following that, topics such as analysis of selection pressure, codon usage, amino acid frequencies, repeated sequences, and reverse repeat contraction and expansion are covered. The Orchidaceae share similar cp chromosomal characteristics, and we have conducted a preliminary analysis of their evolutionary connections. The cp genome of this family has a typical tepartite structure and a high degree of consistency across species. Platanthera urceolata with more tandem repeats of the cp genome. Similar cp chromosomal traits can be seen in the orchidaceae. *Galearis roborowskyi, Neottianthe cucullata, Neottianthe monophylla, Platanthera urceolata* and *Ponerorchis compacta* are the closest cousins, according to phylogenetic study.

## Introduction

The Orchidaceae, which contains 700 genera and 20,000–35,000 species, is the biggest angiosperm family in the world. It is widespread throughout all terrestrial environments, particularly in the tropics, with the exception of arctic and exceptionally dry deserts. In China, there are 171 genera, 1247 species, and subgenera of those species.Due to their distinctive morphology and ecological adaptations, orchids have a high scientific worth in addition to their high economic value. The natural populations of many orchids in China have declined rapidly and are now in danger as a result of the expansion of orchids there and the deterioration of the ecological environment in recent years^1^. There has been a lot of research on orchids in recent years^2^, particularly on their morphology and therapeutic potential, but little has been done on their genetics^3,4^.

As of July 30, 2022, At the National Center for Biotechnology Information (National center for biotechnology information, NCBI) uploaded a total of 1 228 complete chloroplast genome data for Orchidaceae species, Among them, 153 were *Dendrobium* Sw., There are 54 *Paphiopedilum* Pfitz.. There are 28 *Phalaenopsis* Bl., *Dendrobium officinale*, *Paphiopedilum emersonii, Phalaenopsis aphrodite, Cymbidium dayanum, Apostasia ramifera, Bletilla formosana*. The whole chloroplast genome of some orchid species has been published and studied by scholars^5^.In order to determine the evolutionary time and genome position of various ndh gene deletion, Lin^6^ of the chloroplast genome of eight kinds of orchid, found that *Vanilla shenzhenica, Vanilla planifolia, Galeola faberi* and *Drakaea elastica* truncation or absence of ndh gene, the phenomenon has nothing to do with the known taxonomic or evolutionary relationship.

The Orchidaceae is a complicated evolutionary family, but up until now, inadequate research on its affinities and lack of information of its DNA have made it difficult to analyze.Due to their short sequences, uniparental inheritance, low nucleotide substitution rates, and straightforward, conserved genome structure, chloroplast genes were found to be better suited to the study of genetic relationships in plants, or phylogenetic relationships, after being examined from various angles.

Chloroplasts are crucial organelles for photosynthesis in the plant body and are descended from ancient bacteria that were formerly part of early plants and symbionts like cyanobacteria, which enable plants to contribute positively to Earth’s ecosystem^7^. The chloroplast is a very significant and active organelle that performs many different metabolic processes, including photosynthesis. The chloroplast genome evolves more slowly^8^; unlike the frequent changes and recombination in the mitochondrial genome, the single-parent genetic characteristics of the chloroplast genome can effectively reduce the interference of genetic recombination^9^, so the chloroplast genome becomes an appropriate and effective tool for plant classification and phylogenetic research.

A large single copy (LSC) region and a small single copy (SSC) region are separated by two inverted repeat regions in the chloroplast genome's circular DNA structure^10,11^.Chloroplast genomes have been used in phylogenetic and kinship studies between plants recently, which may clearly depict and display their evolutionary ties^12^. In this study, the nine species with the highest species richness—*Neottianthe monophylla, Herminium monorchis, Galearis roborowskyi, Ponerorchis chusua, Platanthera urceolata, Malaxis monophyllos, Ponerorchis compacta, Neottia puberula, Neottianthe cucullata*—had their cp genomes sequenced, assembled, and Additionally, we carried out a thorough evolutionary analysis on the cp genomes of 19 species belonging to the Orchidaceae subfamily.

## Materials and methods

### Sample collection, DNA extraction, and sequencing

Nine species of healthy, fresh leaves (*Neottianthe monophylla, Herminium monorchis, Galearis roborowskyi, Ponerorchis chusua, Platanthera urceolata, Malaxis monophyllos, Ponerorchis compacta, Neottia puberula, Neottianthe cucullata*) were gathered in the Tibet Autonomous Region and Qinghai Province, then quickly frozen and stored in liquid nitrogen. Through the use of a modified cetyltrimethylammonium bromide (CTAB) procedure^13^, whole genome DNA was isolated from leaves.The Illumina NovaSeq 6000 platform was used for the sequencing of all DNA sequences. Over 5000 times the coverage of each complete cp genome was provided by the clean reads.Experimental research and field studies on plants including the collection of plant material are comply with relevant guidelines and regulation.The selected materials were identified by professor Yang Zeng of Qinghai Normal University as *Ponerorchis chusua* and *Ponerorchis compacta*. Plant collection has been granted permission.

### Genome assembly and annotations

Using NOVOPlastyv4.2^14^, we assembled the chloroplast genome, and the more comprehensive results served as the final genome for screening. In addition to manual checks, the newly assembled chloroplast genomes were annotated with the help of PGA (Plastid Genome Annotator)^15^, the cp genome of *Dipterocarpus turbinatus* (Genbank: NC_046842.1) served as a reference, and the tRNA genes were verified with the help of ARAGORNv1.2.38^16^ and tRNAscan-SEv2.0.7^17^. Using OGDRAW, a completely annotated circular plastid map was created (OrganellarGenomeDRAW)^18^.

Using the online MIcroSAtellite (MISA) 2.1 tool, simple repeat sequences (SSRs) were found in the chloroplast genomes of 19 plant species. There were eight single nucleotides, five dinucleotides, four trinucleotides, and three each of tetranucleotides, pentanucleotides, and hexanucleotides utilized as repeat unit parameters.

### Comparative analyses

Taking the *Galearis roborowskyi* genome as a reference, the homogeneity of the entire chloroplast genomes of 19 species were visualized to examine chloroplast genomic differences, using the shuffle-LAGAN program of mVISTA v2.0^19^. Using IRSCOPE, the borders of the IR, SSC, and LSC areas were also compared.DnaSPv6.12.03, which was used to explore nucleotide diversity (Pi), with the window length set to 600 bp and the step size set to 200 bp, was utilized to extract and analyze the coding and non-coding sections.

The codon adaptation index (CAI), codon bias index (CBI), frequency of optimum codons (Fop), effective number of codons (ENc), GC content of synonymous third codon positions (GC3s), and relative synonymous codon usage values were used to evaluate codon preferences (RSCU)^20^.

### Selective pressure analysis

The selection pressure in orchids was calculated using the ratio of non-synonymous substitutions (Ka) to synonymous substitutions (Ks) (Ka/Ks).Initially, the four chloroplast genomes of orchids were compared, and 79 protein-coding genes were extracted. The non-synonymous substitution rate (Ka) and synonymous substitution rate (Ks) for each gene were calculated, as well as the ratio of the two (Ka/Ks), with positive selection being indicated by a Ka/Ks ratio greater than 1, neutral selection being indicated by a Ka/Ks ratio of 1, and purifying selection by a Ka/Ks ratio less than 1.

### Phylogenetic inference

For phylogenetic reconstruction, we collected 10 publically accessible chloroplast genome sequences from the Genbank website (two of which were from external populations). Prior to trimming the lines of comparison for each gene, all single-copy genes from the 19 taxa were extracted. For phylogenetic investigations, they were also joined in a permutational manner.Finally, a phylogenetic tree (ML) was built using TreeBeSTv1.9.2^21^. The support of branches was evaluated by 100 rapid bootstrap replications.

## Results

### Comparison among chloroplast genomic features in Orchidaceae

**Figure 1:**
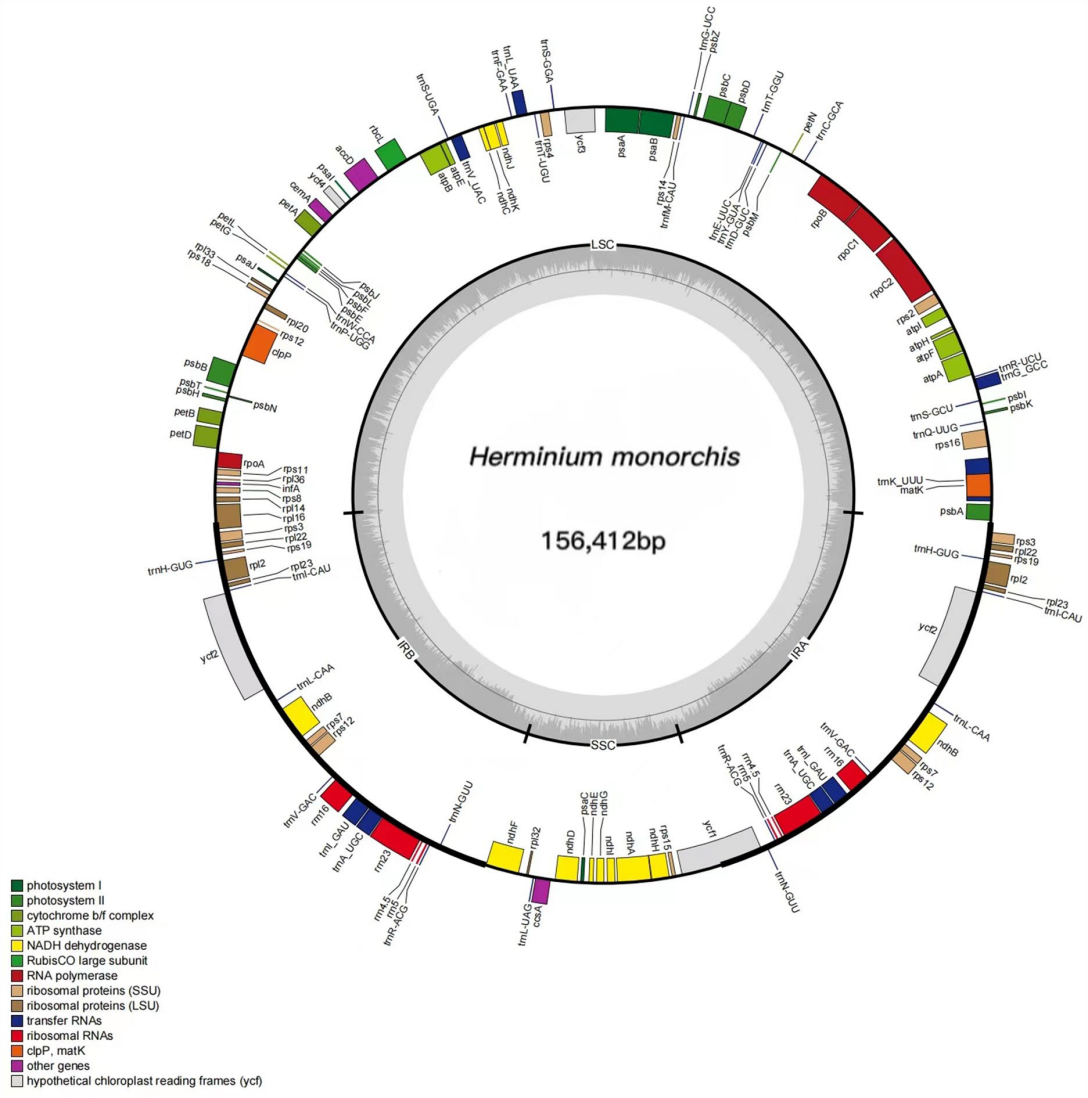
A genetic map of *Herminium monorchis* chloroplast genome. Genes inside the circle have their transcription going clockwise, whereas those outside the circle have it going the other way. Different colors that code for genes designate various functional groupings. The amount of guanine-cytosine (GC) is represented by the grey-black portion of the inner circle, while the amount of adenine–thymine (AT) is shown by the light grey portion. The inner circle displays the reverse repeat (IRa, IRb) regions as well as the small single-copy region (SSC) and large single-copy region (LSC).

*Galearis roborowsky*i was discovered to have the least chloroplast genome size (149,067 bp), whereas *Herminiumm onorchis* had the greatest (156,412 bp) (Fig. 1) (support document). The LSC, SSC, and IR lengths for these 11 species are displayed in Table 1. We discovered between 124 and 140 distinct genes, including between 83 and 94 protein-coding genes, 8 rRNA genes, and 38 tRNA genes (Tables 1, 2).

**Table 1:** Characteristics of the chloroplast genomes of nine species of orchids.

Taxon	Size (bp)	LSC length (bp)	SSC length(bp)	IR length (bp)	GC content			
					Total	LSC	SSC	IR
*Neottianthe monophylla*	153,043	82,786	17,579	26,339	36.7	34.3	29.2	43.0
*Herminium monorchis*	156,412	81,717	15,322	29,687	36.7	34.7	29.9	41.3
*Galearis roborowskyi*	149,067	82,394	15,283	25,695	36.7	34.3	28.6	43.2
*Ponerorchis chusua*	152,852	82,041	17,611	26,600	36.7	34.4	28.6	43.0
*Platanthera urceolata*	154,495	84,072	15,933	27,245	36.4	33.8	28.7	42.5
*Malaxis monophyllos*	151,635	82,843	17,502	25,645	36,7	34.4	29.3	43.1
*Ponerorchis compacta*	154,597	83,744	17,361	26,746	36.4	33.9	28.8	42.8
*Neottia puberula*	153,024	84,487	15,354	26,596	37.6	35.4	30.7	43.1
*Neottianthe cucullata*	154,847	84,137	17,906	26,402	36.5	34.0	28.8	43.0

**Table 2:** Genes difference of the chloroplast genomes of eleven Orchidaceae species.

Taxon	Number of genes	Protein-coding genes	rRNA genes	tRNA genes
*Neottianthe monophylla*	124	83	8	33
*Herminium monorchis*	134	88	8	38
*Galearis roborowskyi*	140	94	8	38
*Ponerorchis chusua*	135	89	8	38
*Platanthera urceolata*	138	92	8	38
*Malaxis monophyllos*	136	90	8	38
*Ponerorchis compacta*	132	86	8	38
*Neottia puberula*	134	88	8	38
*Neottianthe cucullata*	133	87	8	38

**Figure 2:**
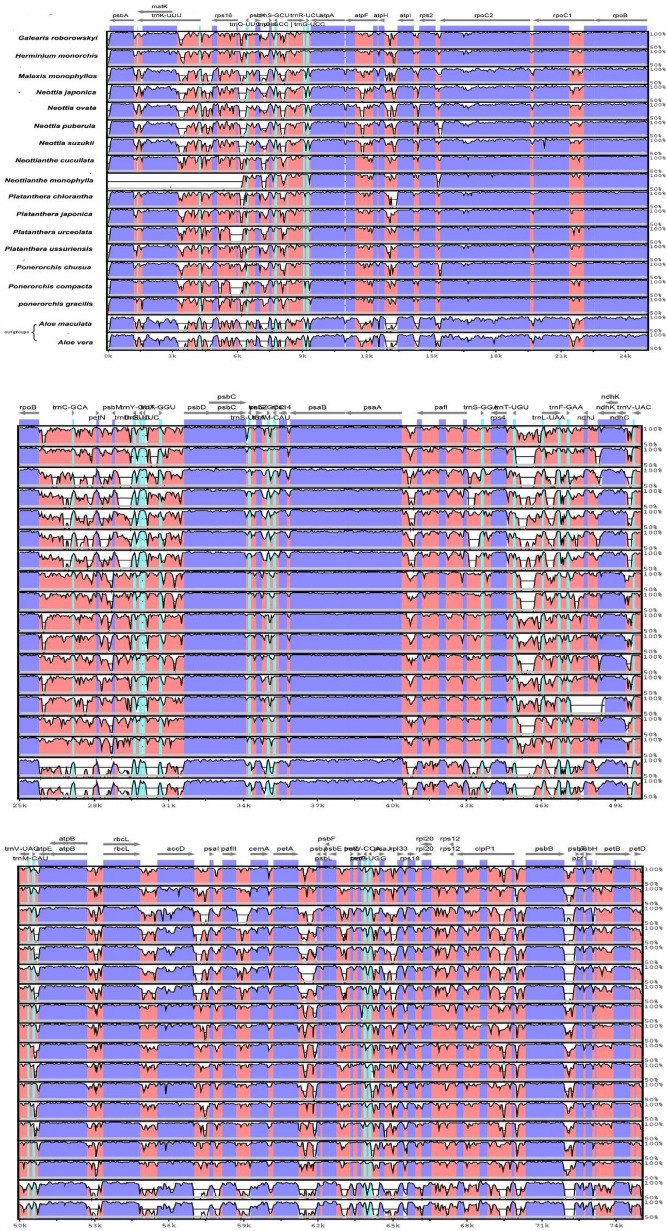

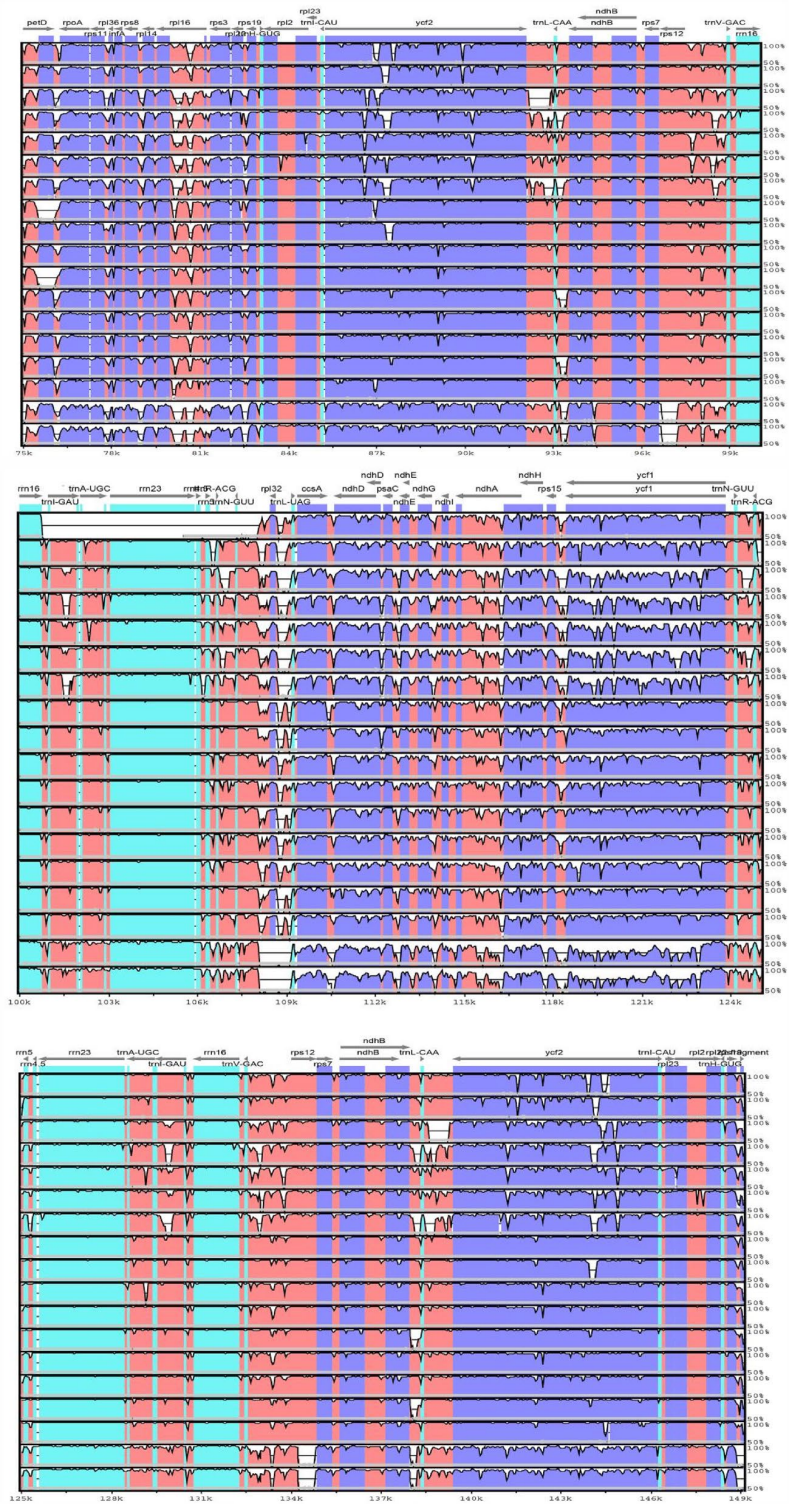
Using the shufe-LAGAN program, the chloroplast genomes of 23 distinct species were examined. The horizontal axis displays the location in the chloroplast genome, and the same proportions are displayed in the vertical direction at a scale of 50 to 100%.The gene being labeled and the direction of transcription are represented by each arrow. Exons, tRNA, conserved non-coding sequences, and mRNA are designated by different colors as genomic areas (support document).

The cp genome of *Galearis roborowskyi* was then further compared using the mVISTA method, revealing a similar pattern of sequences throughout the chloroplast genome, which comprises 17 orchid species and two outgroups (*Aloe maculata* and *Aloe vera*) (Fig. 2, support document).The findings demonstrate that, in contrast to the two outgroups, the chloroplast genomes of 17 species of orchid have comparable architecture and gene sequences. All studied species had more conserved coding regions than non-coding regions. The LSC region also diverged from the SSC region more so than it did from the IR region. The alignment revealed that genes, particularly the *psbA matK* and *rps16* genes, were less conserved in the genomes of *Neottianthe monophylla*.

**Figure 3:**
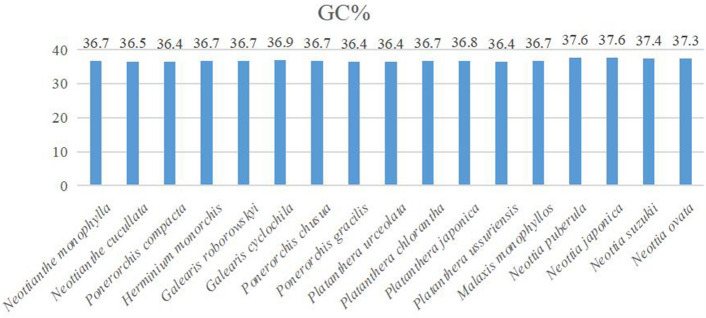
Changes in chloroplast GC content of all 17 species.

Guanine-cytosine (GC) content in orchids ranged from 29.8% (*Platanthera* *contigua*) to 48.1%. (*Orchis militaris*). GC content in the LSC, SSC and IR regions was 33.8–35.4%, 28.6–30.7% and 41.3–43.2%, respectively, Compared to LSC and SSC, the GC content in IR was substantially higher (Table 1; Fig. 3).

### Divergence hotspots

**Figure 4:**
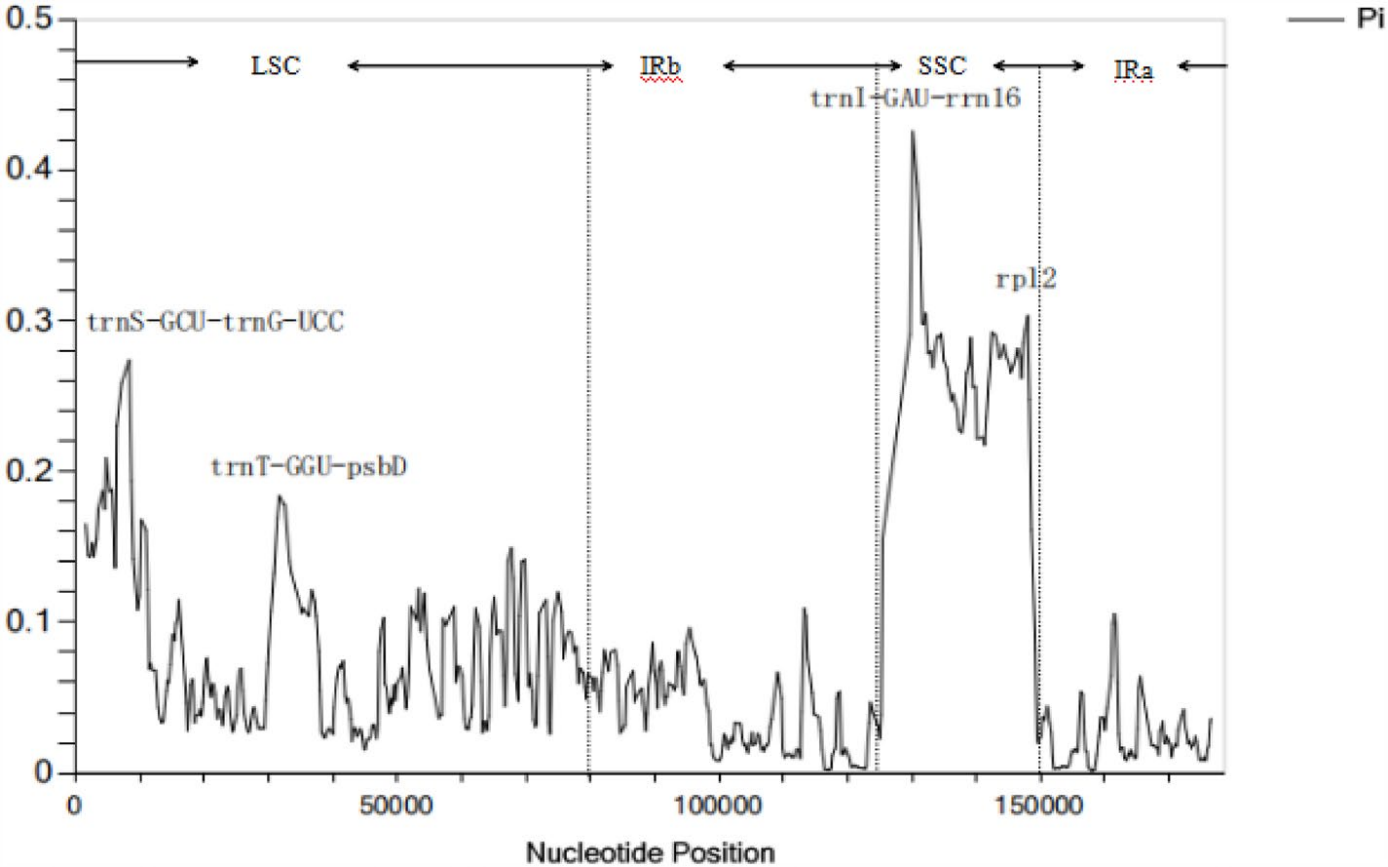
Nucleotide polymorphism of the chloroplast genomes of Orchidaceae species.

DnaSPv6.12.03 was used to analyze the whole cp genomes of 17 orchid species and two Peripheral species. At a window length of 600 bp, the nucleotide diversity (Pi) between sequences was determined and analyzed. There were 623 mutant sites in the aligned gene sequences, with Pi values ranging from 0.00051 to 0.42575 and a mean value of 0.06999. With Pi values over 0.16, four extremely variable locations were found. *TrnS-GCU-trnG-UCC*, *trnT-GU-psbD*, *trnI-GAU-rrn16*, and *rpl2* are some of these regions. The LSC area contains two of these gene fragments, while the IR region contains the other two.*Rpl2* is among them, whereas the rest are found in the non-coding region. Compared to the non-coding area, the genes that code for proteins are more conserved. The region of *trnI-GAU-rrn16* included the difference with the highest value, which was 0.42575. (Fig. 4).

### Contraction and expansion of inverted repeats

**Figure 5:**
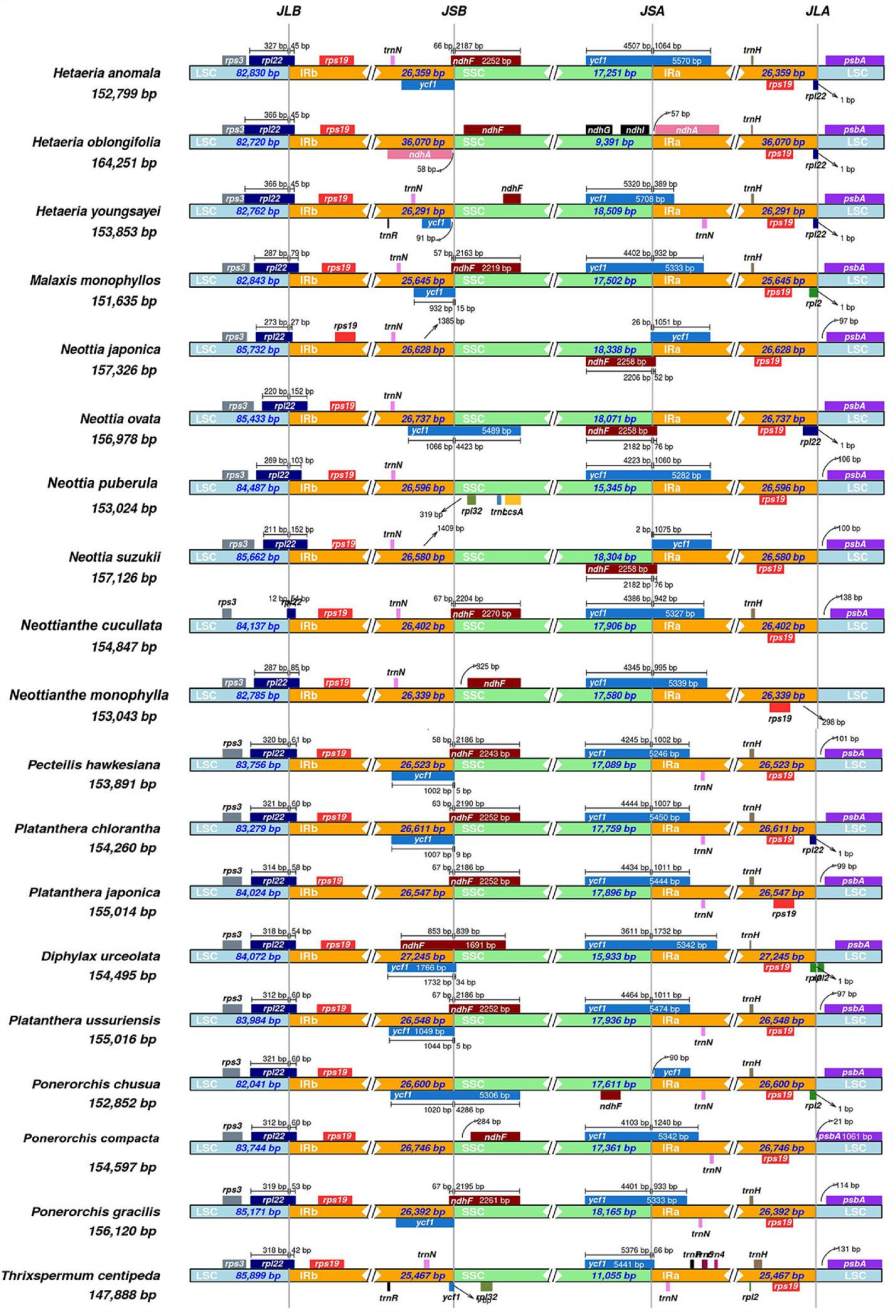

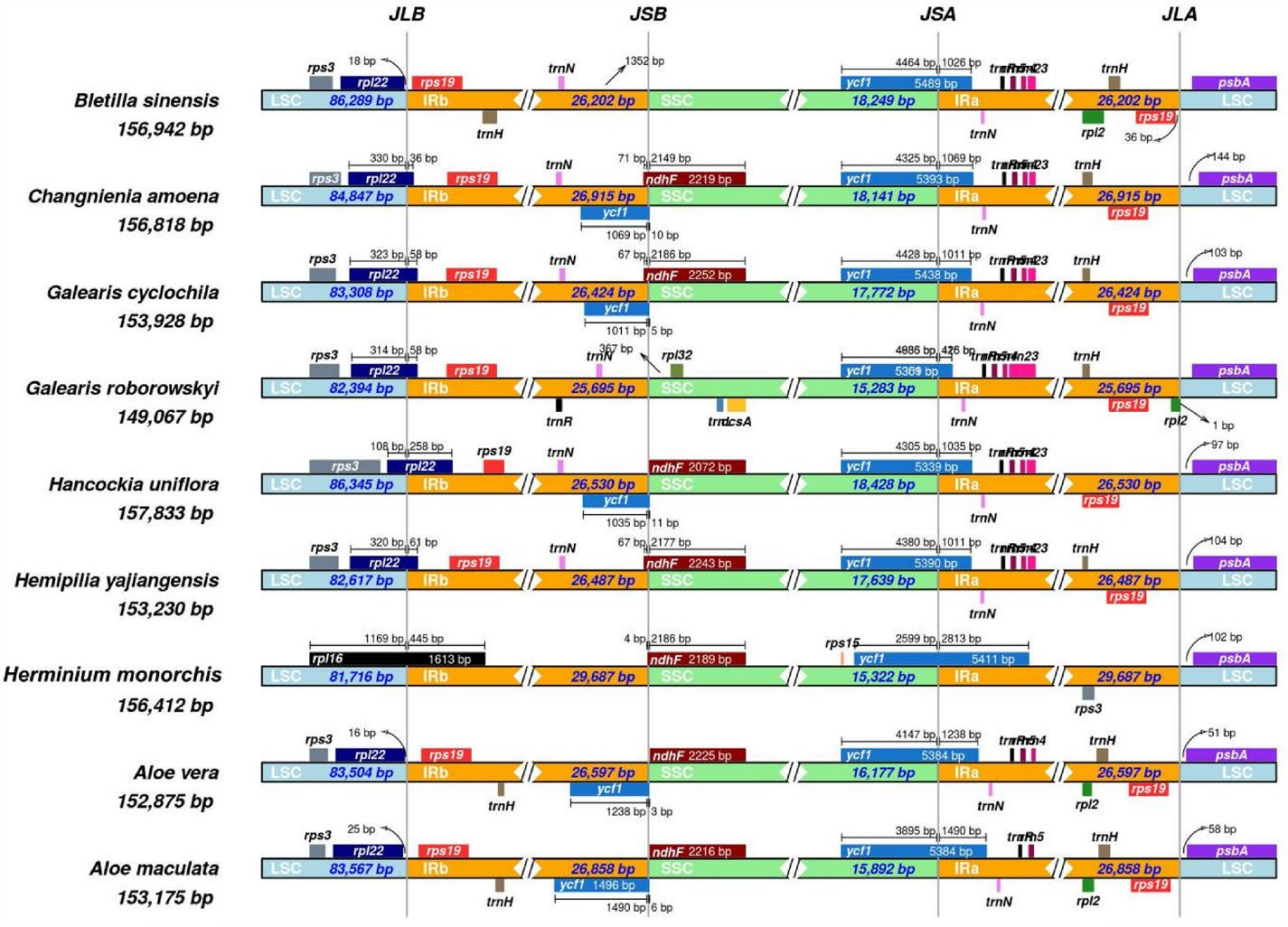
Comparison of the borders of the all regions among 28 chloroplast genomes of Orchidaceae.

The size of the cp genome can alter due to the contraction and growth of the IR area, which also has an impact on the rate at which cp genes evolve^22,23^. A comparison of the IR boundaries of 28 species of orchids revealed that the IR boundary locations with the most pronounced alterations were IRb/SSC, SSC/IRa, and IRa/LSC (Fig. 5).The LSC/IRa and LSC/IRb edges of the Orchidaceae chloroplast genome are substantially conserved, with virtually the same genes flanking them. The *rps3* gene is found on IRb at the junction of LSC and IRb. The *rpl22* gene spans the LSC/IRb area in most species, with *Neottia suzukii* and *Neottia ovata* having the highest expansion, with the exception of *Aloe vera*、 *Aloe maculata* and *Hancockia uniflora*.

### Codon usage and amino acid frequency

The degree of relative synonymous codon usage, sometimes referred to as codon usage preference, measures how frequently a specific codon is utilized in the codon that codes the corresponding amino acid. When RSCU > 1, the codon is used more frequently. This codon has no preference in the case of RSCU = 1; if it is used very infrequently, RSCU1 (support document).

To assess codon usage in the Orchidaceae, we analyzed codon usage deviations for genes in the cp genomes of nine orchid species (Tables 3 and 4). Codon use deviations were derived using relative synonymous codon usage (RSCU). Six codons that encode the amino acids arginine (Arg), leucine (Leu), and serine (Ser) were shown to have the highest preference in this investigation. The arginine was determined to have the highest (2.00 to 2.20) and lowest (0.40 to 0.46 RSCU levels). Other RSCU-related metrics, such as the codon adaptation index (CAI), the codon utilisation index (CBI), the optimal codon frequency (Fop), the effective codon number (ENc), and the synonymous codon 3 position (GC3s), were more moderate.

**Table 3:** The indexes of the codon usage bias of protein-coding genes of Orchidaceae.

	CAI	CBI	Fop	ENc	GC3s
*Neottianthe monophylla*	0.158	− 0.100	0.354	55.13	0.353
*Herminium monorchis*	0.169	− 0.068	0.375	55.00	0.372
*Galearis roborowskyi*	0.160	− 0.100	0.355	54.89	0.350
*Ponerorchis chusua*	0.161	− 0.093	0.359	55.29	0.358
*Platanthera urceolata*	0.160	− 0.105	0.351	54.85	0.351
*Malaxis monophyllos*	0.158	− 0.102	0.352	54.94	0.346
*Ponerorchis compacta*	0.160	− 0.091	0.357	55.15	0.354
*Neottia puberula*	0.158	− 0.086	0.359	55.41	0.364
*Neottianthe cucullata*	0.159	− 0.091	0.359	54.89	0.357

**Table 4:** Codon content of 20 amino acids and stop codons in *Neottianthe cucullata*.

*Neottianthe monophylla*							
AA	Codons	Numbers	RSCU	AA	Codons	Numbers	RSCU
Phe	UUU	2226	0.94	Ser	UCU	1190	1.55
	UUC	2506	1.06		UCC	866	1.13
Tyr	UAU	1496	1.39		UCA	881	1.15
	UAC	650	0.61		UCG	505	0.66
Leu	UUA	1032	1.25	Cys	UGU	666	1.18
	UUG	1015	1.23		UGC	466	0.82
	CUU	1079	1.30	TER	UGA	1005	1.04
	CUC	691	0.84	Trp	UGG	643	1.00
	CUA	681	0.82	TER	UAA	1132	1.17
	CUG	465	0.56		UAG	761	0.79
Pro	CCU	616	1.13	His	CAU	886	1.43
	CCC	528	0.97		CAC	357	0.57
	CCA	714	1.31	Gln	CAA	1018	1.40
	CCG	325	0.60		CAG	436	0.60
Arg	CGU	356	0.71	Ile	AUU	1745	1.19
	CGC	227	0.45		AUC	1118	0.76
	CGA	472	0.94		AUA	1535	1.05
	CGG	296	0.59	Met	AUG	894	1.00
Thr	ACU	687	1.25	Asn	AAU	1723	1.42
	ACC	560	1.02		AAC	709	0.58
	ACA	577	1.05	Lys	AAA	2077	1.37
	ACG	374	0.68		AAG	962	0.63
Ser	AGU	678	0.88	Val	GUU	725	1.33
	AGC	486	0.63		GUC	408	0.75
Arg	AGA	1089	2.17		GUA	655	1.20
	AGG	568	1.13		GUG	397	0.73
Ala	GCU	515	1.38	Gly	GGU	488	0.92
	GCC	339	0.91		GGC	352	0.67
	GCA	437	1.17		GGA	715	1.35
	GCG	197	0.53		GGG	558	1.06
Asp	GAU	990	1.43	Glu	GAA	1304	1.39
	GAC	391	0.57		GAC	574	0.61

### Repeat analyses

**Figure 6:**
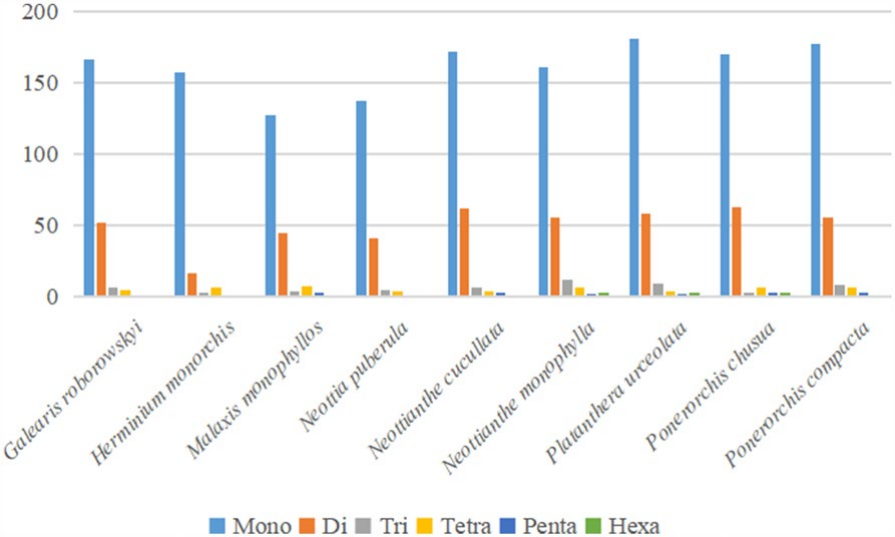
Frequency of diferent microsatellite motifs in diferent repeat types of nine Orchidaceae plastome genomes.

**Figure 7:**
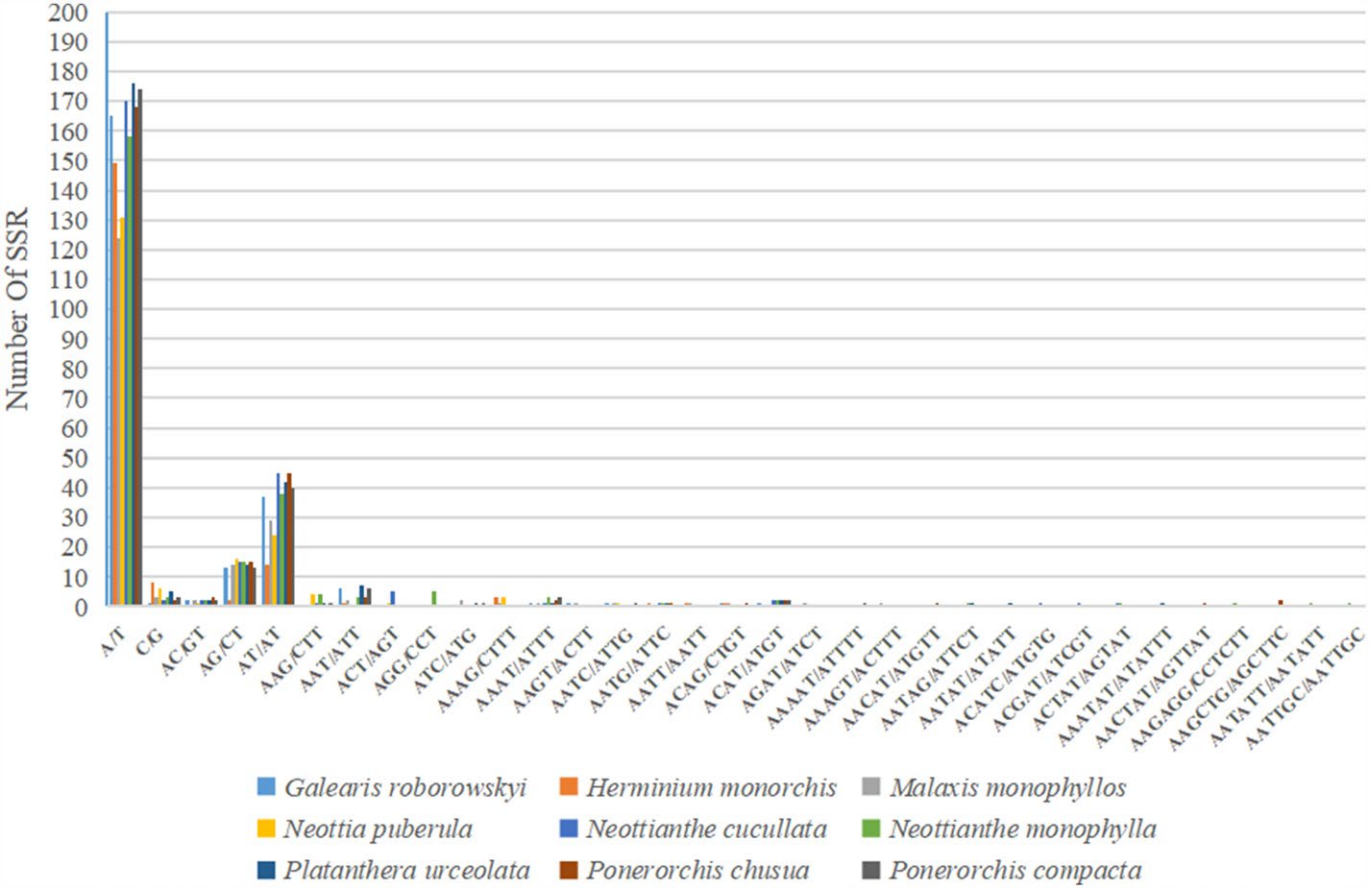
Number of different SSR types in the nine Orchidaceae chloroplast genomes.

*Platanthera urceolata*’s plastid genome had 255 SSRs in total, more than any of the other eight orchid families shown in Fig. 6. The bulk of SSRs in the cp genome (81.9%) were single nucleotides, with poly T and poly A predominating (Figs. 6 and 7). The findings of the analysis of the SSR frequency in the genomes of nine Orchidaceae species are depicted in Fig. 4. All species’ genomes had the dinucleotides AT/AT and AG/CT, however only Herminium monorchis’ dinucleotide AC/GT was absent from the genomes of the other eight species. Furthermore, only *Neottianthe monophylla, Platanthera urceolata*, and *Ponerorchis chusua* were found to have five trinucleotides (AAT/ATT, AAG/CTT, ACT/AGT, AGG/CCT and ATC/ATG), nine tetra-repeats(AAAG/CTTT, AAAT/ATTT, AAGT/ACTT, AATC/ATTG, AATG/ATTC, AATT/AATT, ACAG/CTGT, ACAT/ATGT and AGAT/ATCT), eight pentanucleotides(AAAAT/ATTTT, AAAGT/ACTTT, AACAT/ATGTT, AATAG/ATTCT, AATAT/ATATT, ACATC/ATGTG, ACGAT/ATCGT and ACTAT/AGTAT) and six hexanucleotides(AAATAT/ATATTT, AACTAT/AGTTAT, AAGAGG/CCTCTT, AAGCTG/AGCTTC, AATATT/AATATT, AATTGC/AATTGC) (Fig. 7).

**Table 5:** Number of different SSR types in the nine Orchidaceae chloroplast genomes.

	*Galearis roborowskyi*	*Herminium monorchis*	*Malaxis monophyllos*	*Neottia puberula*	*Neottianthe cucullata*	*Neottianthe monophylla*	*Platanthera urceolata*	*Ponerorchis chusua*	*Ponerorchis compacta*	Total
Mono	166	157	127	137	172	161	181	170	177	1448
Di	52	16	45	41	62	55	58	63	55	447
Tri	6	1	4	5	6	12	9	3	8	54
Tetra	5	6	7	4	4	6	4	6	6	48
Penta	0	0	1	0	3	2	2	1	1	10
Hexa	0	0	0	0	0	3	1	3	0	7
Total	229	180	184	187	247	239	255	246	247	2014

### Selective pressure analysis

**Figure 8:**
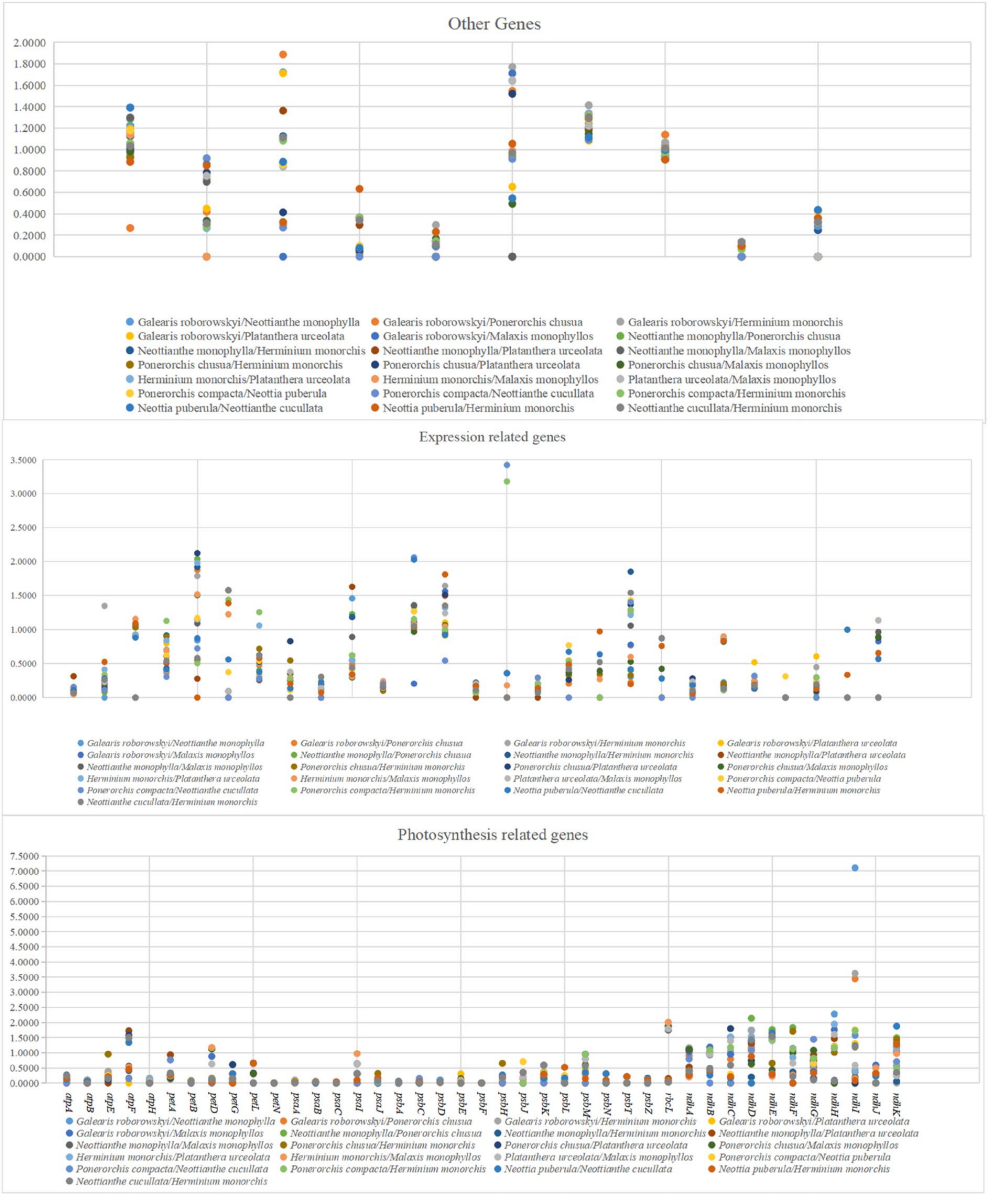
Ka/Ks values of different functional genes.

DnaSP software was used to determine the chloroplast codon Ka/Ks in order to compare the Ka/Ks of 21 distinct species pairs and further examine the selection pressure on chloroplast genes in orchids during evolution (Fig. 8). For each of the 21 species pairs, Ka/Ks ratios were computed. For the pairs *Herminium monorchis-Platanthera urceolata* and *Galearis roborowskyi-Neottianthe monophylla*, higher Ka/Ks ratios were found. The photosynthesis-related genes *atpF, ndhD, ndhE*, and *ndhH,* the expression-related genes *rpl22, rpoC1, rpoC2, accD*, and *ycf1* of other functional genes, and the genes related to expression-related genes *rps18* and *rpoC2* all showed Ka/Ks > 1, indicating that these genes were under positive selection during evolution (Table 5).

### Phylogenetic relationships among Orchidaceae

**Figure 9:**
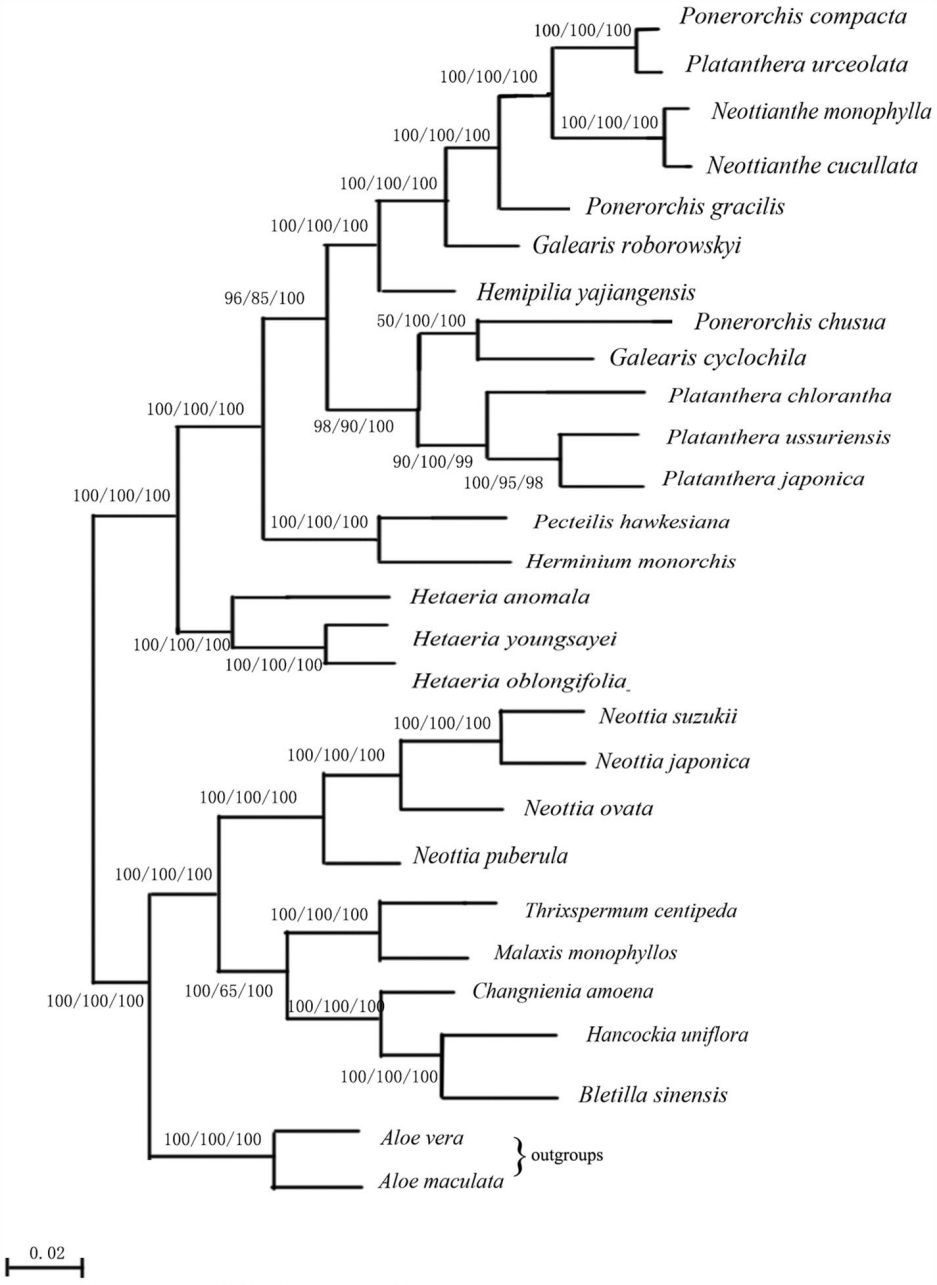
Phylogenetic tree of 28 species based on chloroplast genomes,The topology is indicated with ML/NJ/MP bootstrap support values at each node.

Using *Aloe maculata* and *Aloe vera* as outgroups, the evolutionary relationships of the cp genes in 28 orchid species were investigated. Using the ML、NJ and MP technique, a developmental tree of 50 single-copy genes was created (Fig. 9). The cp genomes of 28 species of orchids were used in this study to infer phylogenetic relationships, and the ML, NJ and MP analysis was used to compare those relationships to outgroups like *Aloe macrophylla* and *Aloe vera*. The tree was created with 26 nodes. All phylogenetic trees have the same topology (the three trees are presented together), and most of the nodes have 100% bootstrap support, indicating high analysis confidence. The 28 orchid species studied are mainly divided into several large clades, of which *Neottianthe, Platanthera, Hetaeria*, and *Neottia* all clearly clustered into one clade, indicating that their congeners are more closely related.

## Discussion

Kim et al. compared the chloroplast genome size of 30 orchids and their gene loss, and found that most of the genes associated in heterotrophic orchids had been lost (ndh), while most of the housekeeping genes retained^24^. This result was also verified in Kim et al.^25^.Chloroplast genomes have strong conservation in plant evolution. First, it is a typical four-segment structure, and then it is highly conserved^26,27^ in gene content and gene order, which is why chloroplast genomes are widely used for phylogenetic research.

Nine Orchidaceae species’ chloroplast genome lengths were examined in this study. Nine Orchidaceae have full chloroplast genomes, with an average length of 153,330 bp and sizes ranging from 149,067 bp (*Galearis roborowskyi*) to 156,412 bp (*Herminiumm onorchis*).The tetrad structure of the chloroplast genome in land plants makes it highly conserved under normal conditions. The majority of the 74 protein-coding genes in the angiosperm chloroplast genome are present, but there are also instances of gene capture, gene rearrangement, and gene loss in various families and species^28,29^. Comparative analysis makes it simple to locate mutation hotspots because of the plant cp genome's highly conserved structure. In population genetic or phylogenetic investigations, these mutational hotspots surrounding by conserved sequences are frequently utilized as DNA barcodes^28,47^. We discovered that sequence variation in Orchidaceae primarily occurs in the non-coding regions via a combined analysis of mVISTA sequence variation and DnaSP-inferred nucleotide variation.Three unique areas (*psbA, matK,* and *rps16*) were identified in the investigation of the sequence variation in the cp genome (Fig. 2). *TrnS-GCU-trnG-UCC, trnT-GGU-psbD, trnI-GAU-rrn16*, and *rpl2* were all shown to be highly variable according to our sliding window analysis. To determine which of these high variation genes or gene spacers could be utilized as accurate and trustworthy DNA barcodes in the genus Orchidaceae, more research is required.

We hypothesize that the nine Orchidaceae species' diverse chloroplast genome lengths may result from the expansion and contraction of the boundary between the SC region and the IR sections^29^. The results further demonstrated the existence of IR areas, as well as their extension and contraction, by comparing and evaluating the IR/SC boundary sections of the nine species of chloroplast genome.The findings demonstrate that all nine Orchidaceae species exhibit the characteristic chloroplast tetrad structure, which is structured in the form of two SC areas and two IR regions at regular intervals.

Mutation pressure^30^ and selection pressure^31^ are the main influencing factors leading to codon preference, but also affected by other factors, such as gene expression level^32^, gene length^33^ and tRNA abundance^34^, etc.Our findings demonstrated that codon usage bias was preserved across species in the Orchidaceae^35^, and that codon usage alterations play a significant role in the evolution of the cp genome. Additionally, the majority of codons preferred to end in A/U with RSCU1, indicating that the cp genome’s adaptive evolution may have contributed to some degenerate codon usage bias^36^. Additionally, all ENc values are greater than 54.85, and the values for CAI, CBI, and Fop are significantly lower than one, showing that all eleven species have very low codon use biases.Liu Jiangfeng selected 47 protein coding sequences from the garlic chloroplast genome of sickle wing, analyzed codon usage patterns, and found that codon preference was affected by selection and mutation, as well as some other influencing factors.

Previous studies showed that polymorphism at the SSR locus is useful in studying population genetics^37–39^.In this study, the minimum repeats of one, two, three, four, five, and six nucleotides were set to 8, 4, 4, 3, 3 and 3 using the MISA software. A total of 255 SSRs, including 226 SSRs made up of the A/T, AT/TA, AAT/ATT, AAAT/ATTT, and AATT/AATT, were found in *Platanthera urceolata.* This confirms that the SSRs in the chloroplast genome are primarily made up of short tandem repeats of the A and T, which is similar to the findings published for other plant chloroplast genomes. Previous research has shown that A/T repeat types predominate among all repeat units in many plant chloroplast genomes, and this phenomenon has also contributed to the extremely high AT content in chloroplast genomes^40^.

Numerous studies have demonstrated that understanding the adaptive genetic evolution of the chloroplast genome is crucial to understanding changes in gene structure and function^41^. It is common practice to utilize the ratio of non-synonymous substitution rates (Ka) to synonymous substitution rates (Ks) as a measure of selection pressure between various species at the sequence level^42^. In most genes in an organism, synonymous nucleotide substitutions occur more frequently than non-synonymous changes; as a result, Ka/Ks values are typically lower than one^43^. This study’s findings indicated that 10 genes had undergone positive selection. *Rpl22, RpoC1, RpoC2*, and *Rps18* were connected to gene expression among these genes.Similarly to the findings of most plant research, the photosynthesis-related genes *atpF, ndhD, ndhE,* and *ndhH* as well as additional functional genes *accD* and *ycf1* all showed Ka/Ks greater than 1 in most species, indicating the presence of positive selection pressure on these genes.

Numerous scientists have conducted in-depth phylogenetic analyses of chloroplast genome sequences in recent years. This is critical to our comprehension of how angiosperms evolved from other organisms^44,45^. Complete chloroplast genomes have been utilized by several researchers to examine the evolutionary relationships and relatedness of plants^46,47^. For this study, 17 published orchid chloroplast sequences were chosen in order to better understand the evolutionary links among orchids. The phylogenetic relationships of orchids were modeled using *Aloe vera* and *Aloe maculata* as outgroups.The findings suggest that the species were split into two groups. *Galearis roborowskyi, Neottianthe cucullata, Neottianthe monophylla, Platanthera urceolata* and *Ponerorchis compacta* all belonged to the same group, and the clustering map made it evident how closely related the Orchidaceae species are to one another. This study gives some theoretical support for the detailed investigation of the phylogeny of orchids and demonstrates the effectiveness of the chloroplast genome in separating out the phylogenetic links of orchid species. In this paper, nine complete orchid chloroplast genomes are revealed.

## Conclusions

An examination of the cp genome sequences of 17 species of orchids revealed that their cp genome organization, gene sequencing, codon use, and repetitive sequence traits are remarkably comparable.In general, these structures are conserved, although the constriction and infrared regions are observed for the expansion of this region associated with plastid sequence variation. Analysis of positive selection of genes in the chloroplast genome of this family suggests that *atpF, ndhD, ndhE,* and *ndhH* may play a role in the growth of most Orchaceae species to strongly light environments.These genomic data provide new insights into the interspecific relationships of the Orchidaceae species. The phylogenetic analysis of the chloroplast genome’s single-copy genes revealed that 19 species may be separated into two groups, which offers some theoretical support for a thorough investigation of the phylogeny of the orchidaceae. This study sets the foundation for further exploration of the taxonomic, phylogenetic and evolutionary history of Orchidaceae.

## Data Availability

Further questions can be sent to the respective authors, whose original contributions are mentioned in the article/supplementary material. The chloroplast genome sequence mentioning species has been uploaded to NCBI with accession numbers shown below:

**Table:** 

	Species	Accession number
1	*Galearis roborowskyi*	PRJNA997927
2	*Ponerorchis compacta*	PRJNA998340
3	*Neottia puberula*	PRJNA998753
4	*Neottianthe cucullata*	PRJNA999017
5	*Ponerorchis chusua*	PRJNA999044
6	*Herminium monorchis*	PRJNA999615
7	*Platanthera urceolata*	PRJNA999050
8	*Neottianthe monophylla*	PRJNA999948
9	*Malaxis monophyllos*	PRJNA999172

The sequence of other closely related and outer related species used in the analysis were downloaded from NCBI with the following accession numbers:

**Table:** 

	Species	Accession number
1	*Aloe maculata*	NC_035505.1
2	*Aloe vera*	NC_035506.1
3	*Bletilla sinensis*	NC_060362.1
4	*Changnienia amoena*	NC_045402.1
5	*Galearis cyclochila*	NC_046818.1
6	*Hancockia uniflora*	OK012601.1
7	*Hemipilia yajiangensis*	NC_067080.1
8	*Hetaeria anomala*	MW589524.1
9	*Hetaeria oblongifolia*	MW589525.1
10	*Hetaeria youngsayei*	MW589526.1
11	*Neottia japonica*	NC_041446.1
12	*Neottia ovata*	NC_030712.1
13	*Neottia suzukii*	NC_041447.1
14	*Pecteilis hawkesiana*	NC_082102.1
15	*Platanthera chlorantha*	NC_044626.1
16	*Platanthera japonica*	NC_037440.2
17	*Platanthera ussuriensis*	MN686021.1
18	*Ponerorchis gracilis*	NC_046810.1
19	*Thrixspermum centipeda*	NC_054174.1
